# Age and Vascular Burden Determinants of Cortical Hemodynamics Underlying Verbal Fluency

**DOI:** 10.1371/journal.pone.0138863

**Published:** 2015-09-22

**Authors:** Sebastian Heinzel, Florian G. Metzger, Ann-Christine Ehlis, Robert Korell, Ahmed Alboji, Florian B. Haeussinger, Isabel Wurster, Kathrin Brockmann, Ulrike Suenkel, Gerhard W. Eschweiler, Walter Maetzler, Daniela Berg, Andreas J. Fallgatter

**Affiliations:** 1 Department of Neurodegeneration, Hertie Institute for Clinical Brain Research (HIH), University of Tuebingen, Tuebingen, Germany; 2 Department of Psychiatry and Psychotherapy, University of Tuebingen, Tuebingen, Germany; 3 German Center for Neurodegenerative Diseases (DZNE), Tuebingen, Germany; 4 Geriatric Center, University Hospital Tübingen, Tübingen, Germany; University Zurich, SWITZERLAND

## Abstract

**Background:**

Aging processes and several vascular burden factors have been shown to increase the risk of dementia including Alzheimer's disease. While pathological alterations in dementia precede diagnosis by many years, reorganization of brain processing might temporarily delay cognitive decline. We hypothesized that in healthy elderly individuals both age-related neural and vascular factors known to be related to the development of dementia impact functional cortical hemodynamics during increased cognitive demands.

**Methods:**

Vascular burden factors and cortical functional hemodynamics during verbal fluency were assessed in 1052 non-demented elderly individuals (51 to 83 years; cross-sectional data of the longitudinal TREND study) using functional near-infrared spectroscopy (fNIRS). The prediction of functional hemodynamic responses by age in multiple regressions and the impact of single and cumulative vascular burden factors including hypertension, diabetes, obesity, smoking and atherosclerosis were investigated.

**Results:**

Replicating and extending previous findings we could show that increasing age predicted functional hemodynamics to be increased in right prefrontal and bilateral parietal cortex, and decreased in bilateral inferior frontal junction during phonological fluency. Cumulative vascular burden factors, with hypertension in particular, decreased left inferior frontal junction hemodynamic responses during phonological fluency. However, age and vascular burden factors showed no statistical interaction on functional hemodynamics.

**Conclusion:**

Based on these findings, one might hypothesize that increased fronto-parietal processing may represent age-related compensatory reorganization during increased cognitive demands. Vascular burden factors, such as hypertension, may contribute to regional cerebral hypoperfusion. These neural and vascular hemodynamic determinants should be investigated longitudinally and combined with other markers to advance the prediction of future cognitive decline and dementia.

## Introduction

Aging as well as neurodegenerative diseases, such as different forms of dementia including Alzheimer's disease (AD), have been associated with vascular, metabolic, structural and functional alterations of the brain [1, 2]. These complex and multifaceted alterations may precede detectable deficits in cognitive performance in the elderly and demented individuals by many years [3–7]. In this regard, reorganization of brain processing and activation has been suggested to partly and temporarily compensate for aging and neurodegenerative processes [8–10]. For instance, age-related upregulation of activation in prefrontal and fronto-parietal networks underlying attention and cognitive control may represent a compensation strategy of cognitive processing in neural circuits [10–12]. Another neural compensation strategy might be a decrease in lateralization of brain activation with increasing age [13]. Thereby, cognitive performance may not differ between elderly and young participants, or cognitive decline as characteristic for mild cognitive impairment (MCI) and dementia might be delayed in the elderly. However, (compensatory) changes in brain processing might represent an early indicator of age-related and neurodegenerative processes, and combined with other risk factors of neurodegeneration further predictive value of future cognitive decline might be provided.

Vascular burden factors represent important risk factors of neurodegeneration contributing to pathomechanisms of dementia and cognitive decline via their impact on neural and hemodynamic functions. (1) Chronic brain hypoperfusion leading to cerebral hypoxia has been shown to have profound effects on neural functions and to represent one important etiological factor of neurodegenerative processes in AD and vascular dementia [14–16]. (2) Many vascular burden factors including hypertension, diabetes, obesity, hypercholesterolemia, smoking or stroke have been shown to increase the risk of dementia [16–21] and the conversion from MCI to AD [22].

Difficulties in effortful word retrieval during verbal fluency are among the earliest signs of dementia [23]. Phonological and semantic verbal fluency has been shown to elicit cortical activation, i.e. hemodynamic responses within prefrontal, fronto-temporal and parietal regions [10, 24–26]. Previously, we showed in a cohort of 325 non-demented elderly individuals that increasing age predicts increased hemodynamic correlates of fronto-parietal and decreased inferior frontal junction activation during verbal fluency [10].

Using cross-sectional data of the largest functional near-infrared spectroscopy (fNIRS) study so far, the present study first aimed at replicating these previous findings of age-related reorganization during verbal fluency in a large sample of non-demented elderly individuals (TREND study). Second, the impact of vascular burden factors on functional hemodynamic responses during verbal fluency processing was investigated. Third, a possible interaction between age and vascular burden factors on these hemodynamic responses was analyzed. Thus, the present cross-sectional study aimed to provide age-related vascular/neural markers, which may be validated as predictors of cognitive decline as investigated in future longitudinal analyses of the TREND study.

## Methods

### 2.1 Participants

In total, 1102 non-demented elderly individuals (age: 51–84 years) participated in the TREND study (**T**übinger evaluation of **R**isk factors for **E**arly detection of **N**euro**D**egeneration; first follow-up, March 2011—April 2012, see: http://www.trend-studie.de/english/ and [10, 27–30]). Analyses of fNIRS and vascular burden factor data varied in sample size due to differences in data availability or analysis strategy. 1) fNIRS data (n = 1052): Data of 50 individuals were excluded from fNIRS analyses due to technical problems or missing data (n = 20), excessive motion artifacts as identified by visual inspection (n = 17), or non-German mother language (n = 13) as important for verbal fluency performance. Since a first strategy was to replicate previous findings (n = 325) of age-effects on fNIRS signals, an independent sample including the remaining 727 individuals was established. 2) Vascular burden factors and fNIRS: For analyses of effects of vascular burden factors on fNIRS signals the entire fNIRS sample (n = 1052) was investigated; however, data of vascular burden factors of 49 individuals (smoking history, n = 40; body-mass-index, n = 9) were not available. This resulted in differences in sample size regarding analyses of the number of vascular burden factors (n = 1003) and analyses of the single vascular burden factors.

### 2.2 Hemodynamic measurements during verbal fluency

We used fNIRS recordings to assess cortical hemodynamic responses elicited during the performance of phonological and semantic verbal fluency as previously described [10, 25]. Briefly, changes in relative light absorbance corresponding to changes in concentration of oxygenated (oxy-Hb) and reduced hemoglobin (deoxy-Hb), respectively, were measured. The attenuation of light absorbance is related to hemodynamic changes along an ellipsoid pathway through scalp, skull and cortical tissue between a light emitter and a detector [31]. We used an array of emitter-detectors (3-cm distance) to record hemodynamic responses from two probe-sets comprising a total of 44 channels (ch). The probe sets were adjusted using head surface position markers of the international 10–20 system and covered bilateral prefrontal, parietal and frontotemporal cortex regions. For the left probe-set the optode between left ch 1 and 2 was positioned on T3, and for the right side the corresponding contralateral optode was positioned on T4. The most caudal row of optodes was adjusted on a horizontal line of Fpz-T3 and Fpz-T4, respectively. Based on these spatial specifications channels-wise anatomical information were derived using probabilistic anatomical labeling [32]. The estimation error (standard deviation) of the projection of channels onto the cortical surface of the standard brain is indicated by the size of circles.

To account for possible systemic artifacts, e.g. arousal and increases in blood pressure and heart rate, the global signal of all channels was subtracted from the single channel's signal at each sampling point (common average reference correction) [10, 33]. Thus, reported hemodynamic responses of single channels are relative to the global hemodynamic response. Oxy-Hb has been suggested to represent a more sensitive and reliable indicator of changes in regional cerebral blood flow compared to deoxy-Hb, and as in our previous study [10] oxy-Hb data was thus the primary dependent variable of the fNIRS data [34–36]. The oxy-Hb time-series signal was averaged over respective verbal fluency task conditions (30-s blocks each: phonological task: words starting with the letter: A/F/M; semantic task: words of the category: professions/fruits/flowers; control task: weekdays from Monday to Sunday). The magnitude of task-related hemodynamic response was indicated by the mean amplitude of each channel. Lateralization magnitude was assessed by subtracting the right channels' hemodynamic response amplitude from the corresponding contralateral left channel.

### 2.3 Vascular burden factors

We previously identified vascular factors predicting increased carotid intima-media thickness independent of age and sex, thus indicating vascular burden effects in the TREND cohort [37]. The identified vascular burden factors include diabetes mellitus (diabetes; life-time medical diagnosis, intake of antidiabetic medication or HbA_1c_ levels ≥ 6.5), arterial hypertension (hypertension; life-time medical diagnosis), obesity (body mass index > 30 kg/m^2^), smoking (> 15 pack-years) and atherosclerosis (life-time medical diagnosis). Assessment of these factors involved questionnaires and personal medical history interviews.

### 2.4 Statistical analysis

First, to replicate previous findings of aging-related changes of cortical hemodynamic changes during verbal fluency, fNIRS and statistical analyses were analogously performed in an independent sample of 727 individuals as published (n = 325; [10]). Accordingly, multiple regression analyses of channel-wise hemodynamic response amplitudes (oxy-Hb; Letter-Weekday (phonological) or Category-Weekday (semantic)) as criterion variable and the predictors (inclusion algorithm) age, sex, years of education and task performance were conducted. The prediction of the lateralization magnitude by age was investigated accordingly. To account for the multiple testing situation, the significance threshold was adjusted using false-discovery-rate (FDR) corrections [38]. As indicator of the size of effect the standardized β-weight of the predictor in the multiple regression is reported.

Second, we analyzed the prediction of vascular burden factors on hemodynamic response of the channel showing the highest response amplitude (peak channel). Therefore, we additionally included the number of vascular burden factors (0, 1, 2+) in the regression models (also comprising the other predictors reported above). We then tested the single vascular burden factors accordingly for an prediction of the peak channel response. For display purposes, mean values and standard error of hemodynamic response amplitudes of the vascular burden factor groups were, in the respective figure, adjusted for the influence of covariates (i.e., the other predictors in regressions) using analyses of covariance.

Third, interaction effects between vascular burden factors and age impacting the peak hemodynamic response amplitude were analyzed by also entering an interaction term in the regression models. Here, centered values of age and the dummy variable hypertension, their product (the interaction term), as well as sex, years of education and task performance were entered as predictors in the regression model.

The significance threshold was set to α = 5%. The fNIRS data were analyzed using custom routines in Matlab 2009b (The MathWorks, Natick, MA, USA). Statistical analyses were performed using IBM SPSS 22 (SPSS, Inc., Chicago, IL, USA).

## Results

### 3.1 Descriptive statistics

Overall (n = 1052), the mean age of individuals was 65.2 ± 6.8 years (mean ± standard deviation; range: 50.8–83.7 years) with females (64.3 ± 6.9 years; n = 504) being younger than males (66.1 ± 6.7 years; n = 548; t_1050_ = -4.3, p < .001). Females obtained shorter formal education (13.0 ± 2.8 years) than males (14.6 ± 2.6 years; t_1050_ = -9.2, p < .001). Within blocks of 30-s participants pronounced on average 6.1 ± 2.0 correct words in the phonological, and 9.7 ± 2.0 correct words in the semantic verbal fluency task condition.

The independent sub-sample (n = 727; 302 females, 425 males) had a lower percentage of females than the rest of the sample (n = 325; 202 females, 123 males; χ^2^ = 38.2, p < .001). However, age, years of education, and phonological and semantic verbal fluency performance did not differ between sub-samples (p > .05).

Predefined vascular burden factors including hypertension (41% of 1052; n = 431), diabetes (9.4% of 1052; n = 99), obesity (13% of 1043; n = 140), smoking more than 15 pack-years (14.2% of 1012; n = 149), and atherosclerosis (5.1% of 1052; n = 54) were considered for analyses of single vascular factors. Groups of individuals without vascular burden factors (44.7%; n = 448 of 1003), with one (35.6%; n = 357), or with two or more (19.7%; n = 198) vascular burden factors were defined.

### 3.2 Hemodynamic responses elicited by verbal fluency

**Fig 1:**
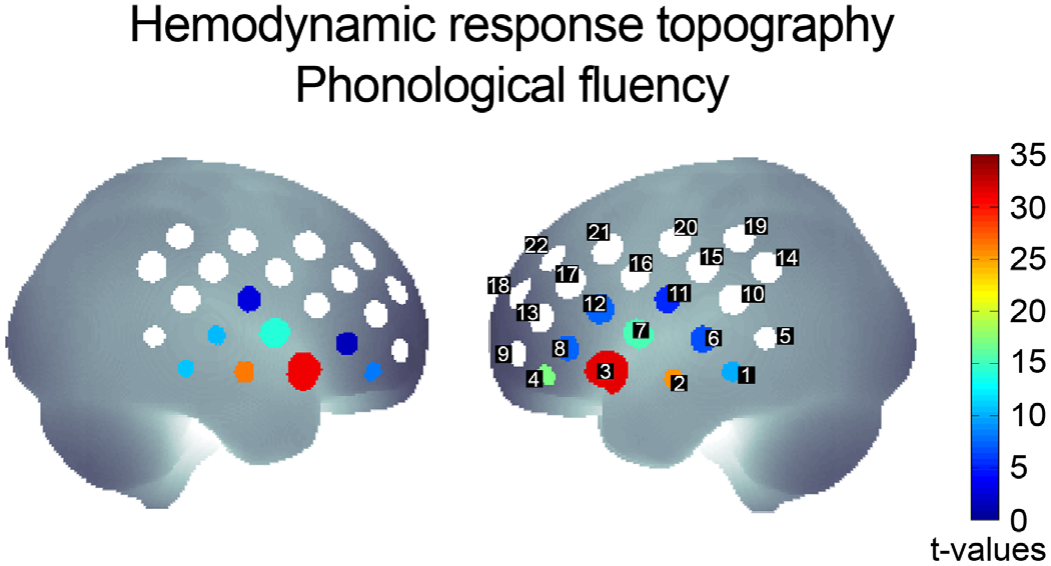
Topography of significantly increased hemodynamic responses during phonological fluency compared to the control condition (FDR-corrected) in the total sample. Channel numbers of the left hemisphere correspond to contralateral channel numbers.

Phonological as well as semantic verbal fluency elicited strong hemodynamic responses compared to the control task. Responses were most pronounced in bilateral inferior frontal junction (peak channel 3, left) and fronto-temporal areas (Fig 1), where phonological compared to the semantic task conditions elicited stronger responses in both hemispheres (ch 3, left: t_1051_ = 14.5, p < .001; right: t_1051_ = 12.1, p < .001). The peak latency within the 30-s task blocks did not differ between phonological (23.2 ± 6.3 s) and semantic fluency (23.4 ± 5.6 s; p > .1).

For phonological fluency significant lateralization effects (p < .05, FDR-corrected) were observed within the inferior frontal gyrus (left > right, ch 4, 8, 12; peak ch 4: t_1051_ = 7.50, p < .001) as well as in the postcentral gyrus (right > left, ch 20) and middle temporal gyrus (right > left, ch 2, 6; peak ch 6: t_1051_ = -4.14, p < .001). For semantic fluency lateralization was not as pronounced with inferior frontal (left > right, ch 4, 8, peak ch 4: t_1051_ = 6.83, p < .001) and middle temporal gyrus (right > left, ch 6; t_1051_ = -2.87, p < .001) exhibiting significant hemispheric differences (p < .05, FDR-corrected).

### 3.3 Age effects on hemodynamic responses during verbal fluency

**Fig 2:**
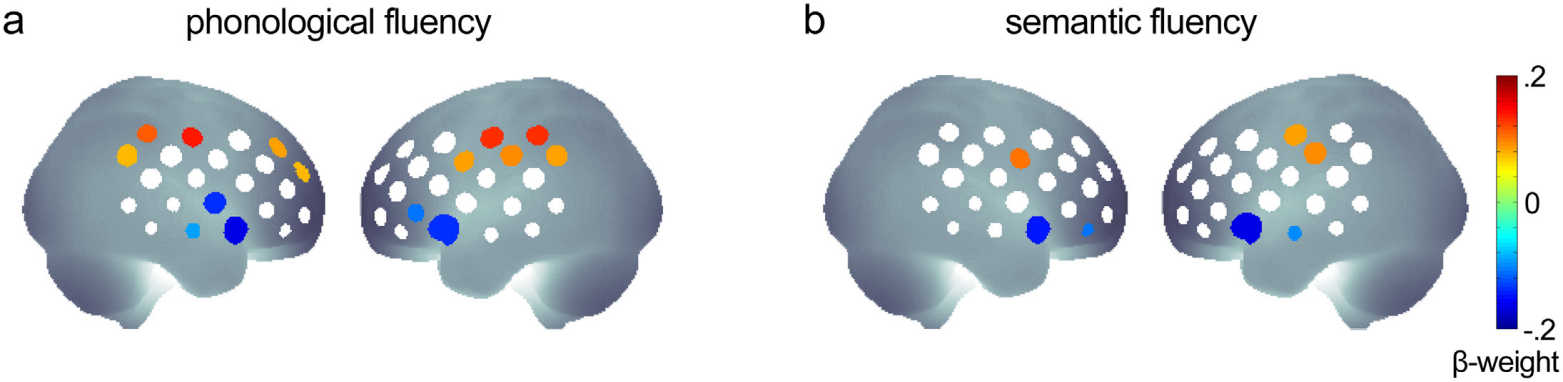
Standardized β-weights of the predictor age in multiple regression analyses of hemodynamic responses in the replication sample of 727 individuals (a/b) and the initial sample (c/d; n = 325). Channels with significant (FDR-corrected) prediction of hemodynamic responses by age during (a) phonological fluency, and (b) semantic fluency. Regression models included the predictors age, sex, years of education and task performance.

In the replication sample (n = 727), age significantly predicted increased hemodynamic responses during phonological fluency in right prefrontal (middle frontal gyrus) and bilateral inferior parietal regions (supramarginal gyri) extending towards postcentral gyri. At the same time, age predicted decreased hemodynamic responses in bilateral fronto-temporal areas (inferior frontal junction) (Fig 2a). For semantic fluency, age only predicted decreased hemodynamic responses in bilateral fronto-temporal areas but not increased responses in middle frontal gyrus or inferior parietal regions (Fig 2b). All age effects were small as indicated by standardized β-weights of -0.2 < β < 0.2.

Lateralization of hemodynamic responses (left > right: inferior frontal gyrus) during verbal fluency showed a non-significant statistical trend toward prediction of decreased lateralization with increasing age (ch 8; β = -.06, p = .053, uncorrected).

### 3.4 Effects of sex, education and task performance

In addition to age other variables showed significant prediction (FDR-corrected) in the channel-wise regression models of hemodynamic responses during verbal fluency. During phonological fluency (n = 727), females showed higher hemodynamic responses compared to males in bilateral inferior frontal junction (peak effect: ch 3; left: β = -.181, p < .001; right:-.185, p < .001; FDR-corrected), whereas males exhibited higher hemodynamic responses than females in right middle frontal gyrus (ch 21; β = .136, p < .001; FDR-corrected). For the semantic fluency condition sex was no significant predictor of hemodynamic responses. Years of education predicted increased right middle frontal gyrus responses for both phonological (ch 22; β = -.131, p = .002, FDR-corrected) and semantic fluency task conditions (ch 21; β = -.127, p = .002, FDR-corrected). In the regression models of each channel none of the hemodynamic responses was significantly (uncorrected) predicted by the respective verbal fluency task performance.

### 3.5 Impact of vascular burden factors

**Fig 3:**
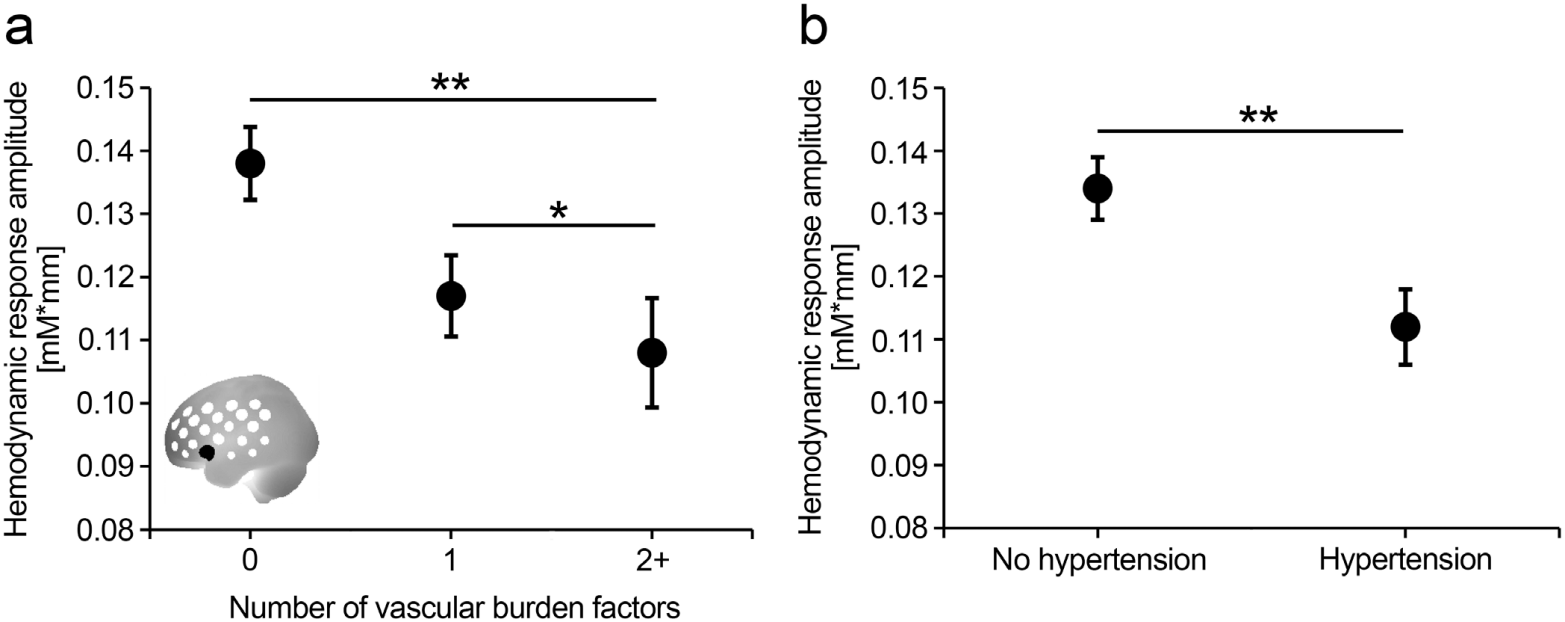
Mean hemodynamic response amplitudes (left channel 3, indicated in black, left inferior frontal junction) with standard errors of the mean in the peak activation region during phonological fluency. Mean amplitudes were adjusted for the covariates age, sex, education and performance of mean amplitudes. Values are shown for a) individuals without, with one, or with two or more vascular burden factors, and b) for individuals without and with hypertension.

In the total sample, an increasing number of vascular burden factors significantly predicted a decrease in hemodynamic response amplitude in the peak activation region (ch 3, left inferior frontal junction) during phonological fluency (β = -.097, p = .002). Here, the additional predictors age (β = -.150, p < .001), sex (β = -.220, p < .001), education (β = .083, p = .016) and task performance (β = .041, p = .210) were also entered in the regression model. Vascular burden group differences in left IFJ hemodynamic responses adjusted for the influence of age, sex, education and performance are shown in Fig 3a.

However, this effect of vascular burden factors was largely due to hypertension (β = -.086, p = .005), which was the only significant single vascular burden factor predicting the decreased left IFJ hemodynamic response. Individuals with hypertension (n = 431) showed decreased hemodynamic response amplitude compared to those without (n = 621; Fig 3b).

For the semantic fluency task, the number of vascular burden factors showed no significant prediction (p > .1), while hypertension (β = -.053, p = .084) showed a statistical trend towards a prediction of decreased left IFJ hemodynamic response amplitudes.

The global hemodynamic response amplitude, i.e. mean of all channels, during phonological or semantic fluency was not impacted by single or the cumulative number of vascular burden factors (p > .1).

### 3.6 Age and hypertension

**Fig 4:**
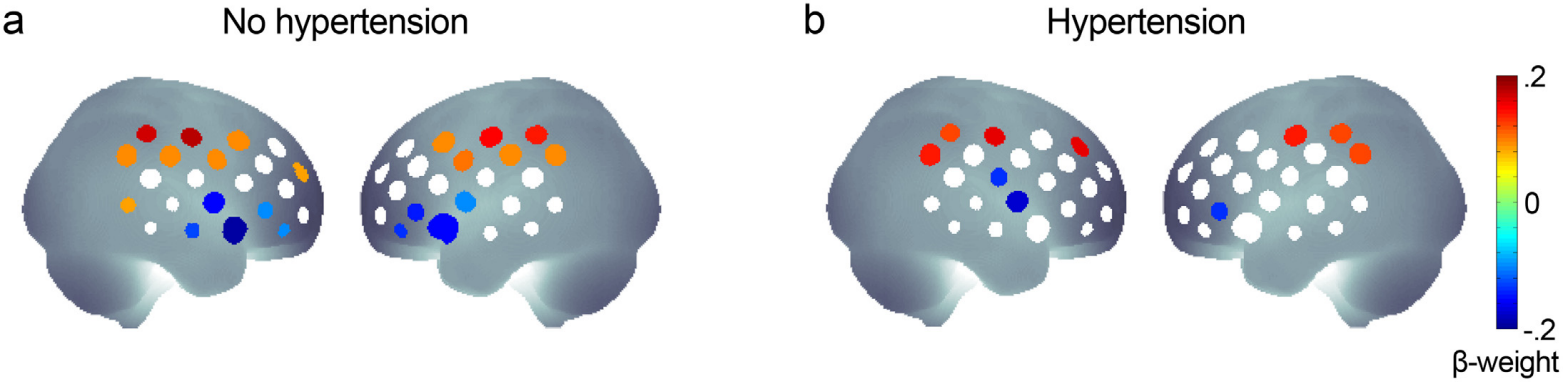
Standardized β-weights of the predictor age in channel-wise (FDR-corrected) multiple regression analyses of hemodynamic response amplitudes during phonological fluency in a) individuals without hypertension, and b) individuals with hypertension. Regression models included the predictors age, sex, years of education and task performance. Interaction analyses of factor age and hypertension were not significant.

Differences in age-related reorganization of cortical processing during verbal fluency between individuals with and without hypertension were investigated (Fig 4). Age and hypertension were tested for an interaction on the peak hemodynamic response (phonological fluency, left IFJ, ch 3). However, the interaction variable entered in the regression did not significantly predict hemodynamic responses in the left IFJ (p > .1). Also, including the interaction term did not improve the fit of the regression model to the hemodynamic response data.

## Discussion

The present study provides further evidence of age-related reorganization of cortical processing during verbal fluency. Replicating previous findings [10], this largest fNIRS study so far showed that increasing age is a predictor for increased hemodynamic response within right prefrontal (middle frontal gyrus), bilateral inferior parietal regions (supramarginal gyri) and postcentral gyri, while at the same time predicting decreased responses in bilateral inferior frontal junction (IFJ). Here, magnitude and topography of the association between age and NIRS activation during verbal fluency was highly comparable to our previous study (n = 325) [10]. Moreover, the number of vascular burden factors, with hypertension in particular, predicted decreased hemodynamic responses in left IFJ.

Dorsolateral prefrontal and inferior parietal regions are part of a superordinate fronto-cingulo-parietal cognitive control network, which is involved in executive domains, such as flexibility, inhibition, initiation, and working memory [39]. Increased activation with increasing age in these areas during demanding cognitive tasks, such as phonological verbal fluency, may thus reflect increased recruitment of attention, executive and cognitive resources. This may indicate aspects of a neural basis of cognitive reserve [9], however whether compensatory reorganization might delay cognitive decline due to age/neurodegenerative processes or increase resilience of some individuals needs is still unclear. In contrast to age-related compensatory reorganization or overrecruitment, an alternative interpretation of increased activation with increasing age may be based on the concept of neural dedifferentiation. Accordingly, increasing age might be accompanied with a loss in functional specificity and/or regional specialization of neural processing [40, 41]. Moreover, the overactivation and underactivation often observed in older compared to younger adults may be interpreted on the basis of the compensation-related utilization of neural circuits hypothesis (CRUNCH) [42]. According to the CRUNCH model, in order to meet task demands at relatively low cognitive loads older adults may increase frontal or bilateral neural engagement compared to young adults, who show more focal activation. However, at higher cognitive demands older adults may have already reached limits of neural capacities, thus, showing underactivation and a decline in performance, whereas younger adults show relative overactivation and better performance than older adults. In the present study, the phonemic fluency task performance (which is considered more difficult than semantic fluency) was not correlated with age, while in the semantic fluency condition increasing age was associated with a slight decline in performance (partial correlation corrected for sex: r = .13, p < .001). Thus, in terms of cognitive load this differential association is in contrast with the CRUNCH model positing an increase, and not decrease, in age-related task performance differences with task difficulty. However, phonemic and semantic fluency may differ in behavioral strategies and neural processing complicating direct comparisons of performance and neural effects between these conditions. Possibly, the lack of age-related differences in the phonemic fluency condition may suggest a compensatory overactivation (e.g. frontal) rather than dedifferentiation of activation. Semantic fluency, however, may to a greater extent require temporal cortex processing resources [43] and age-related performance differences could not be neurally compensated through the observed age-related increase in activation in semantic fluency.

Here, for both task conditions longitudinal analyses should investigate whether the inability to reorganize neural processing or the loss in functional specificity, respectively, in specific individuals may reflect an increased risk of future cognitive decline.

However, effect sizes of age-effects on hemodynamic responses were small (-0.2 < β < 0.2). Therefore, large sample sizes may be required to detect this effect and other putative predictors of cognitive decline should, in combination with the presented factors, also be considered to improve prediction models of cognitive decline. Thus, the neural/vascular correlates of cognitive processing with small effect will need to be integrated into prediction models of cognitive decline which collate different markers of small to intermediate effects to provide a valid and reliable detection of individuals at risk of neurodegeneration. Here, fNIRS as a stand-alone marker might not be adequate. Also, we did not find any association between hemodynamic responses elicited by the verbal fluency tasks and task performance indicated by the number of correct words when accounting for age, sex and education. Thus, the relationship between neural/hemodynamic correlates of verbal fluency and task performance/behavior/strategies needs to be further investigated. Since this lack of association might partly be due vascular/neural [44], anatomical [31] and task strategic differences [45] between individuals, longitudinal intraindividual changes might show a closer association between neural/vascular and cognitive indices of verbal fluency. In addition to alterations in neural processing, the reliable supply of oxygen and glucose to the brain might be insufficient in individuals with increased vascular burden; particularly in regions and situations of increased metabolic demands, e.g. neural regions highly engaged in cognitive task processing. Thus, decreased functional hemodynamic responses in the left IFJ, the region exhibiting the peak response during verbal fluency processing, observed in individuals with vascular burden factors may indicate a critical vascular and neural situation preceding cognitive decline and dementia. While the effect size was small (β = -.097), the impact of the number of vascular burden factors (hypertension, diabetes, obesity, smoking, atherosclerosis), with hypertension in particular, on hemodynamic responses within left IFJ complements previous positron emission tomography findings. Glucose hypometabolism in the left IFJ was shown in patients with early dementia compared to healthy individuals, which was correlated with executive performance during verbal fluency [46, 47]. Moreover, glucose hypometabolism in the left IFJ has recently been shown for healthy elderly (mean: 70 years) compared with young individuals (29 years), which was correlated with decreased structural white matter fiber integrity in short fibers to the prefrontal cortex as well as in long association fronto-temporo-occipital fibers [1]. Thus, the present finding converges with previous evidence suggesting that the left IFJ is a region highly susceptible for vascular burden factors reducing functional hemodynamic responses, i.e. the supply of oxygen and glucose to this region. This impact may contribute to very early pathological processes underlying structural alterations and cognitive decline preceding neurodegenerative diseases, such as AD or other forms of dementia. Such vascular etiological aspects of dementia, with AD in particular, may also converge with β-amyloid pathomechanisms as suggested by amyloid depositions in vascular compartments, and associations between β-amyloid burden and arterial stiffness in elderly individuals without dementia [48]. Also, cardiorespiratory fitness has been shown to mediate the effects of age on cerebral blood flow which was negatively correlated with blood pressure [49]. Possibly, improving vascular health may be a mechanism through which exercise and fitness prevents cognitive decline.

Individuals with hypertension showed a similar fronto-parietal topography and no statistical difference regarding the predictive value of age for the hemodynamic responses compared to individuals without. Hypertension and age did not significantly interact in the regression models of the hemodynamic responses. Thus, the age-related reorganization of processing may not be modulated by vascular burden factors, and both measures of neural reorganization as well as vascular functions might be independently integrated into prediction models of future cognitive decline and dementia.

As in our previous study, also the extended sample showed a non-significant statistical trend of age as a predictor of reduced lateralization (left > right) in the inferior frontal gyrus. Alterations in activation lateralization as compensation strategy have been suggested by the HAROLD model [13], which was derived from findings related to memory and inhibitory processes, but specifically from verbal fluency data. As verbal fluency elicited bihemispheric fronto-temporal activation, age-related compensation by decreasing lateralization might not be efficient. The observed (non-significant) lateralization effects might therefore be modest in the present study. However, conclusions from the present lateralization findings have to be cautiously drawn, since compared fNIRS channels of the two hemispheres might differ in the path-lengths of the fNIRS light and scull-cortex distances.

Sex effects predicting of hemodynamic responses during phonological fluency were consistent with previous findings showing higher hemodynamic response amplitudes in females compared to males in bilateral inferior frontal junction, while males exhibited higher hemodynamic responses than females in right middle frontal gyrus [10]. Elderly females compared with males have been shown to employ more switching strategies (compared to clustering within word categories) in phonological as well as semantic fluency [45]. Thus, differences in activation might result from different performance strategies.

Both age-related reorganization as well as effects of vascular burden factors were more pronounced for the more difficult phonological compared to the semantic verbal fluency condition. Thus, increased task difficulty and resulting cognitive demands may increase the impact of both age and vascular burden on functional hemodynamic responses. Subjective task difficulty (and possibly the selection of task strategies [45]) might change with increasing cognitive impairment which needs to be considered for neural/vascular predictors of cognitive decline.

### Limitations

Several limitations of the present findings have to be considered. (1) Functional hemodynamic responses as measured using fNIRS or fMRI may only represent a correlate of the underlying neural activity during performance of a given task. Thus, (neuro)vascular functions may be partly related to age-related effects on functional hemodynamic and not neural functions per se. However, we found age to predict both region-dependent increase and decrease, respectively. Also, the impact of vascular burden factors was regionally specific indicating that these factors did not generally affect hemodynamic measurements limiting their interpretation as correlates of neural activity. (2) Changes in skin blood flow have been argued to modulate fNIRS signals elicited by a phonologic verbal fluency [50]. To correct for such systemic artifacts the global fNIRS signal was subtracted from each channel's time-series [10, 33]. Moreover, to eliminate arousal confounders and signal changes related to word production, we contrasted the mean amplitudes of experimental with the control condition, where a comparable number of words were verbally articulated. Thereby, blood flow changes within the skin/muscle masking the functional hemodynamics within the cortical gray matter of interest were hopefully minimized. (3) Individual differences in (neuro)anatomy have been shown to affect fNIRS sensitivity and relative channel positions and might therefore account for some error variance in the fNIRS data [31, 44]. Also, age-related decrease in brain volume and changes in differential path-length [51] may have added error variance to the data and contributed to the reported age-effects. However, by correcting for global hemodynamic responses we aimed to (partially) correct for individual (age-related) cortical atrophy. Also, since with increasing age both increased and decreased hemodynamic responses were observed general age-related cortical atrophy may not underlie the present findings. (4) We only used questionnaires and personal medical history interviews for the assessment of vascular burden factors and their definitions involving diseases and medication data. However, it was emphasized that all indications, diagnoses and medications must have been confirmed by a medical doctor. (5) The present results might not be fully generalizable as TREND study participants were partly selectively included due to the presence of prodromal markers for neurodegeneration (REM-sleep behavior disorder, hyposmia and/or depression).

## Conclusions

Age-related reorganization of verbal fluency processing and vascular burden factors represent regionally specific determinants of cortical functional hemodynamics during increased cognitive demands. Showing only small age-related effect these neural and vascular characteristics determinants should be investigated longitudinally (e.g. in the TREND study) and combined with other markers in order to detect individuals at risk for future cognitive decline and dementia.
